# Reduction of Antibiotic Resistant Bacteria During Conventional and Advanced Wastewater Treatment, and the Disseminated Loads Released to the Environment

**DOI:** 10.3389/fmicb.2018.02599

**Published:** 2018-10-30

**Authors:** Thomas Jäger, Norman Hembach, Christian Elpers, Arne Wieland, Johannes Alexander, Christian Hiller, Gerhard Krauter, Thomas Schwartz

**Affiliations:** ^1^Institute of Functional Interfaces, Karlsruhe Institute of Technology, Eggenstein-Leopoldshafen, Germany; ^2^Aquantec, Gesellschaft für Wasser und Umwelt GmbH, Karlsruhe, Germany; ^3^Xylem Services GmbH, Herford, Germany; ^4^Zweckverband Klärwerk Steinhäule, Neu-Ulm, Germany

**Keywords:** antibiotic resistance, wastewater treatment, ozonation, UV irradiation, DNA damage, qPCR, modeling, daily discharge

## Abstract

The occurrence of new chemical and microbiological contaminants in the aquatic environment has become an issue of increasing environmental concern. Thus, wastewater treatment plants (WWTPs) play an important part in the distribution of so-called new emerging pathogens and antibiotic resistances. Therefore, the daily loads released by the WWTP were calculated including a model system for the distribution of these loads within the receiving water body. UV-, as well as ozone-treatment in separate or in combination for wastewater treatment were under investigation aiming at the reduction of these loads. Here, the impact of these treatments on the DNA integrity via antibody staining and PCR efficiencies experiments were included. All three facultative pathogenic bacteria [enterococci (*23S rRNA*), *Pseudomonas aeruginosa (ecfX*), and *Escherichia coli* (*yccT*)] and seven clinically relevant antibiotic resistance genes (ARGs) (*mecA* (methicillin resistance gene), *ctx-M32* (β- lactame resistance gene), *ermB* (erythromycine resistance gene), *bla*_TEM_ (β- lactame resistance gene), *sul1* (sulfonamide resistance gene), *vanA* (vancomycin resistance gene), and *intI1* (Integrase1 gene) associated with mobile genetic elements were detected in wastewaters. Different reduction efficiencies were analyzed during advanced wastewater treatments. ARGs were still found to be present in the effluents under the parameters of 1.0 g ozone per g dissolved organic carbon (DOC) and 400 J/m^2^, like *ctx-M32, ermB, bla*_TEM_, *sul1*, and *intI1*. Especially UV radiation induced thymidine dimerization which was analyzed via antibody mediated detection in the metagenome of the natural wastewater population. These specific DNA alterations were not observed during ozone treatment and combinations of UV/ozone treatment. The dimerization or potential other DNA alterations during UV treatment might be responsible for a decreased PCR efficiency of the 16S rRNA amplicons (176, 490, and 880 bp fragments) from natural metagenomes compared to the untreated sample. This impact on PCR efficiencies was also observed for the combination of ozone and UV treatment.

## Introduction

Municipal wastewater treatment plants (WWTPs) are already identified as sources of nutrients, inorganic and organic pollutants as well as antibiotic resistant bacteria (ARB) and resistance genes (ARGs) (Guo et al., 2013; Michael et al., 2013; Rizzo et al., 2013; Hembach et al., 2017). Some ARB can be removed through conventional wastewater treatment processes (Guardabassi et al., 2002; Da Costa et al., 2006), but there are still large numbers that survive in the effluent (Pruden et al., 2006; Hembach et al., 2017). As a consequence ARB and ARGs are released and widely distributed in the environment (Kim and Carlson, 2007; Czekalski et al., 2012; Alexander et al., 2015). The hygienic quality of receiving waters affected by WWTP effluents are of high relevance, especially by water reuse. For example, the European Urban Wastewater Treatment Directive (Directive, 1991) advised that “treated wastewater shall be reused whenever appropriate” under the requirement of “minimizing the adverse effect on the environment” which is defined as the protection of the environment from the adverse effects of wastewater discharges. It is important to determine the daily discharges of WWTPs which are released into the receiving waters when it's reused for crop irrigation or used as raw water reservoir. With the goal to interrupt dissemination pathways, advanced technologies have to be identified which are able to reduce the bacterial load and minimize the risk of WWTP effluents for subsequent water reuse or human health.

Therefore, several wastewater treatment options are discussed for their capability to reduce the ARB and ARG in the final effluent of WWTPs to achieve an adequate water quality (Norrby et al., 2009; WHO, 2014; Ventola, 2015). Still, a coherent assessment concept is missing to prove the success of reduction efficiency of microbial parameters. Since ozone is frequently used to remove chemical micro-pollutants (Lee and von Gunten, 2010; Ruel et al., 2011), and UV irradiation was reported to damage nucleic acids in bacterial cells (McKinney and Pruden, 2012) and reduce ARG abundances in wastewater (Munir et al., 2011; Hu et al., 2016), this study tightly focuses on the reduction of antibiotic resistant bacteria during conventional and advanced wastewater treatment. Ozonation is described to be an efficient process to remove organic micro-pollutants and also considered adequate to inactivate bacteria via production of highly reactive radicals (Hollender et al., 2009; Zimmermann et al., 2011; Dodd, 2012; Lüddeke et al., 2015; Zhuang et al., 2015). A previous study reported a selection of a robust bacterial population via ozonation, which is characterized by a high GC-content of their genomes (Alexander et al., 2016). Here, pseudomonads including *P. aeruginosa* containing GC-contents >60% (Lee et al., 2006; Hyatt et al., 2010) were identified as ozone robust. The germicidal effects of UV light is inducing alterations on DNA, RNA, and proteins by absorbing irradiation at the respective wavelength (absorption max. for DNA 260 nm, absorption min. 280 nm) (Jungfer et al., 2007; Süß et al., 2009). UV radiation is also known to accelerate horizontal gene transfer (HGT) (Aminov, 2011) by mobile genetic elements (MGEs), which is considered as the main factor driving resistome alteration in aquatic habitats (Chao et al., 2013). This advanced wastewater treatment technologies induce HRT due to the activation of different repair mechanisms involved in dissemination of ARGs. The present study shows the effect of ozone treatment (1 g ozone per g DOC), UV treatment (400 J/m^2^), and the combination (400 J/m^2^ + 1 g ozone per g DOC) on facultative pathogenic bacteria and ARGs present in the wastewater of a large scale WWTP, as well as the impact of these advanced wastewater treatment technologies on the bacterial DNA integrity. Furthermore, we calculate the daily discharges of facultative pathogenic bacteria and antibiotic resistance genes into the adjacent receiving river and simulate different flow rate scenarios. Modeling approaches illustrate the dispersion of the different targets along the receiving river sides, which might be important for reuse approaches in downstream areas.

## Materials and methods

### Sampling

At a large scale WWTP (440,000 population equivalents; average sewage quantity 112,000 m^3^/day) the inflow, conventionally treated wastewater and the final effluent, as well as advanced technologies using either an UV system apparatus (Collimated Beam Device) designed by the company with a mercury low pressure lamp (254 nm) (NLR2036) (Xylem Services GmbH, Herford, Germany), the ozone system type OCS-GSO30 by WEDECO or a combination of both techniques on conventionally treated wastewater were under investigation. According to the turbidity of the water sample the UV intensity was adjusted to 400 J/m^2^. Ozone treatment was adjusted to 1 g ozone per 1 g DOC according to the dissolved organic carbon and a retention time of ~5 min (flow rate ca. 7 m^3^/h). This ozone concentration was specified by the operation company for further reduction of the organic trace substances of treated wastewater. Grab water samples were taken from the sampling points at four sampling campaigns (09/2016, 03/2017, 07/2017, and 10/2017). The wastewater samples were filtered by vacuum filtration on polycarbonate membranes (Ø 47 mm, pore size 0.2 μm, Whatman Nucleopore Track-Etched Membranes, Sigma-Aldrich, Munich, Germany) using 200 to 250 mL of the water samples. By using propidium mono azid (PMA, 25 μM) prior to DNA extraction according to Jäger et al. (2018), the evaluation of disinfection processes can be limited to viable cells with intact cell membranes and an overestimation by molecular biology methods can be avoided (Nocker et al., 2007a,b). A recent study revealed that PMA treatment in wastewater samples is a suitable tool to focus on the viable part of the population. In this study, the authors were focusing on the indicator bacteria *E. coli* and enterococci and showed no significant differences between the cultivation-based approaches and the PMA-qPCR experiments, but there were significant differences between the culture-based method and qPCR experiments without PMA treatment (Li et al., 2014; Jäger et al., 2018). Possible wastewater matrix effects on the PMA efficiencies should be controlled with internal standard experiments and the PMA concentrations should become adjusted to the wastewater characteristic of state. This was done previously for this study.

### DNA extraction for quantitative PCR analysis

DNA was extracted using the FastDNA^TM^ Spin Kit for soil (MP Biomedicals, Illkirch, France). The membranes of the filtered wastewater samples were directly used for DNA extraction and were placed in the Lysing Matrix E tube for mechanical cell disruption. The further DNA extraction steps were performed following the manufacturer's protocol. The concentration of the extracted DNA was measured by using the Qubit™ 3.0 (Thermo Fisher Scientific, Nidderau, Germany).

### Quantitative PCR analysis

SYBR Green qPCR experiments were performed on the Bio-Rad Cycler CFX96 (CFX96 Touch™ Deep Well Real-Time PCR Detection System, Bio-Rad, Munich, Germany) and the analysis was done using the manufacturer's software (Bio-Rad CFX Manager Software). All samples were measured in technical duplicates by qPCR. The reaction mixture consisted of 1 μL template DNA, 1 μL Primer FW (10 μM), 1 μL Primer Rev (10 μM), 10 μL Maxima SYBR Green/ROX qPCR Master Mix (2X) (Thermo Fisher scientific, Nidderau, Germany). Nuclease-free water (Ambion, Life technologies, Karlsbad, Germany) was added to adjust a total volume of 20 μL. The used thermocycler profile consisted of 1 cycle at 95°C for 10 min for DNA polymerase activation, followed by 40 cycles consisting of 95°C for 10 s, and 60°C for 30 s for primer annealing, and elongation. A melting curve, ranging from 60 to 95°C (0.5°C/s), was performed to confirm the specific amplicon.

Calibration curves were generated using extracted DNA from the different reference bacteria, i.e., facultative pathogenic bacteria carrying the respective resistance gene using the DNA extraction kit for soil (MP Biomedical, Illkrich, France). A regression line was made for each tested gene by using serial dilutions of the extracted DNA of the corresponding reference strain to calculate the gene specific cell equivalents (Hembach et al., 2017; Rocha et al., in press). The primer systems and the calculation of the cell equivalents were done based on the already known genome sizes of the retference bacteria and are listed in Supplementary Information Table 1. The PMA-treatment was performed prior to DNA extraction to consider the viable fraction of the wastewater sample (Jäger et al., 2018). The Ct–values from the wastewater samples were adjusted to the corresponding regression line and then normalized to 100 mL of filtered wastewater to show the different reduction efficiencies of absolute abundance within the surviving population of the wastewater samples.

### Detection of DNA damages via PCR

To analyze DNA damages, extracted DNA originating from the different sampling points were used in PCR experiments to distinguish the polymerase efficiency, as described by Süß et al. (2009). Therefore, different 16S rRNA amplicons (176, 490, and 880 bp) were investigated and afterwards separated by gel electrophoresis to distinguish the light units intensities via a F1 Lumi-Imager workstation (Roche Diagnostics) using the included Lumi-Imager software (LumiAnalyst 3.1). Afterwards the light units were determined and normalized to the control. Therefore, the amplicons were separated by a 2% w/v agarose gel electrophoresis and the light units of each amplicon were determined and normalized to their corresponding amplicon of the untreated wastewater sample so that the control results in a value of 1, and the other values represent the light units of the corresponding band in the agarosegel according to the control band. In each PCR reaction 2.5 μL Buffer (10x), 0.5 μL dNTPs (10 μM), 0.25 μL of each Primer (40 μM), 0.125 μL TaqPolymerase and 1 ng/μL template were used and the volume was adjusted to 25 μL by adding water. The thermoprofile consists of 3 min at 95°C followed by 25-times 95°C for 30 s, 56°C for 1 min, and 72°C for 2 min. The last step was an extended elongation step with 72°C for 7 min. Afterwards the samples were cooled down to 4°C.

### Detection of DNA damages via immunological assay

For the DNA damage analyses with antibodies samples were directly mixed with RNA protect to stop any further degradation of the DNA. As control sample untreated wastewater was used. For further processing the samples were spotted on a positively charged nylon membrane (Roche Diagnostics, Mannheim, Germany) using a slot-blot apparatus (Slot-Blot R Microfiltration Apparatus, Bio-Rad, Munich, Germany) connected to a vacuum pump. Triplicates of each sample were tested using 200 μL per slot. Lysis of the bacterial cells was done directly on the nylon membrane by adding 500 μL of lysing and denaturation solution (1.5M NaCl, 0.5M NaOH, pH 13) and incubated for 20 min. This step was repeated three times. Afterwards the solution was removed by vacuum filtration followed by two neutralization steps with 500 μL neutralization solution [1.5M NaCl, 0.5M Tris/HCl (pH 7.2), 1 mM EDTA (pH 8.0)] Then a washing step with 300 μL TBS (0.5M Tris/HCl, 1.5M NaCl, pH 7.5) was performed. Afterwards the nylon membrane was removed from the apparatus and dried for 15 min on a clean filter paper. The immunoreaction was done in a hybridization tube continuously rotating starting with a blocking reaction with 5% non-fat milk solution at room temperature (RT) for 1 h. This was followed by the binding of the primary antibody (anti-CPD or anti-6–4 PP) 1:2,000 diluted in 5% non-fat milk solution for 30 min at 37°C. The incubation of the secondary antibody was performed at 37°C for 1 h. Two washing steps with TTBS (TBS + 1/100 Tween 20) were performed between the treatments. Afterwards two final washing steps with TBS were performed. In addition to the in the protocol mentioned antibodies anti-CPD or anti-6–4 PP (Cosmo Bio Co., Tokyo, Japan), which is based on Kraft et al. (2011), here, a different secondary antibody IgG-AP (Sigma-Aldrich, Munich, Germany) was used. Before developing the blot with the alkaline phosphatase reagent, the membrane was equilibrated with a detection buffer (0.1M Tris-HCl, 0.1M NaCl, pH 9.5) for 5 min at RT. The chemiluminescence detection (CSPD ready to use, DIC High Prime DNA labeling and detection Starter Kit II, Roche) was done at the F1 Lumi-Imager workstation (Roche Diagnostics) using the Lumi-Imager software (LumiAnalyst 3.1).

### Calculation of daily charges of ARB and ARGs

For the calculation of the daily charges the annual mean discharge of the WWTP was used (1.165 m^3^/s), according to the information by the operator of the WWTP. The obtained qPCR data given in cell equivalents per 100 mL were transformed to cell equivalents per m^3^ and multiplied with 86400 s (24 h) (formula 1).

Formula 1: Calculation of the discharge of the WWTP within 24 h given in cell equivalents/ 24 h.

$$\begin{array}{l} \frac{\text{cell equivalents }}{{\text{m}}^{3}}\;\times \text{ annual mean discharge }\left[\frac{{\text{m}}^{3}}{\text{s}}\;\right]\times 24\text{ h }\left[\text{s}\right]\\ \;=\;\frac{\text{cell equivalents}}{\text{24 h}}\\ \frac{\text{cell equivalents }}{{\text{m}}^{3}}\;\times \;1.165\;\frac{{\text{m}}^{3}}{\text{s}}\;\times 86400\text{ s }\\ \;=\;\frac{\text{cell equivalents}}{\text{24 h}} \end{array}$$

For the calculation of the cell equivalents in the river regarding the dilution factor of different water levels, the formula 2 was used. For the river Danube low water is indicated by a flow rate of 22 m^3^/s, mean water by 124 m^3^/s, and flood water by 994 m^3^/s.

Formula 2: Calculation of the concentration within the river system at different water level scenarios (low water, mean water, and flood water).

$$\begin{array}{l} \;(\frac{\text{cell equivalents }(\text{effluent})}{{\text{m}}^{3}}\;\times \text{ annual mean discharge }\left[\frac{{\text{m}}^{3}}{\text{s}}\;\right])\\ ÷\text{water level }\left[\frac{{\text{m}}^{3}}{\text{s}}\right]=\;\frac{\text{cell equivalents }(\text{river})}{{\text{m}}^{3}}\\ \;\left(\frac{\text{cell equivalents }(\text{effluent})}{{\text{m}}^{3}}\;\times \;1.165\;\frac{{\text{m}}^{3}}{\text{s}}\;\right)÷22\frac{{\text{m}}^{3}}{\text{s}}\;\\ =\;\frac{\text{cell equivalents }(\text{river})}{{\text{m}}^{3}} \end{array}$$

### Modeling of the distribution within the receiving body (river danube)

A steady state and transient hydraulic 2D-water flow model (Hydrodynamic Wave Propagation Model HDWAM) originally developed by the Aquantec GmbH to assess and manage flood risks was used in this study. HDWAM is a one- and two-dimensional hydraulic model. A finite-volume discretization is applied to the diffusive wave equations and an implicit scheme is used for time integration (Krauter, 2002).

HDWAM is extended by a water quality module (GQSM) in order to simulate the dispersal of antibiotic resistance bacteria/ genes (ARB/G). The transport of quality parameters in 2D-compartments in the GQSM is described by the following partial differential equation (formula 3).

Formula 3: Partial differential equation describing the transport of quality parameters in 2D-compartments in the GQSM.

$$\begin{array}{l} \frac{\partial hC_{i}}{\partial t}+\frac{\partial q_{x}C_{i}}{\partial x}-\frac{\partial }{\partial x}\left(hD_{\tau }\frac{\partial C_{i}}{\partial x}\right)+\frac{\partial q_{y}C_{i}}{\partial y}-\frac{\partial }{\partial y}\left(hD_{\tau }\frac{\partial C_{i}}{\partial y}\right)\\ \;-\frac{1}{h}\sum_{j=1}^{nzu}q_{zu,j}C_{zu,j,i}+\frac{C_{i}}{h}\sum_{j=1}^{nab}q_{ab,j}=0 \end{array}$$

*h* water depth [m]
*q*_*x*_ specific flow rate in x-direction [m^2^/s]
*q*_*y*_ specific flow rate in y-direction [m^2^/s]
*C*_*i*_ concentration of quality parameter i [mass/m^3^, C°, …]
*D*_τ_ turbulent dispersion coefficient [m^2^/s]
nzu number of external inflow by coupling
*q*_*zu,j*_ external specific inflow j [m^2^/s]
*C*_*zu,j,i*_ concentration of quality parameters i in external inflow j [mass/m^3^, C°, …]
nab number of external outflow by coupling
*q*_*ab*_ external specific outflow [m^2^/s]

The turbulent viscosity can approximately be determined by the depth-averaged parabolic model (formula 4).

Formula 4: The depth-averaged parabolic model to determine the turbulent viscosity.

$$\begin{array}{l} \mu_{\tau }=c_{\mu }\sqrt{ghI_{E}}h \end{array}$$

*g* Gravitational constant [m/s^2^]

*I*_*E*_ Energy gradient [-]

*c*_μ_ Dimensionless coefficient for characterization of the riverbed [Natural riverbeds are characterized by *c*_μ_ between 0.3 (riverbed with low roughness) and 0.9 (riverbed with high roughness)].

The required finite element mesh (FE-mesh of the 2D-hydraulic model HydroAs-2D) for the part of the Danube River with the WWTP is placed at disposal by courtesy of the water authority Donauwörth (© Wasserwirtschaftsamt Donauwörth, www.wwa-don.bayern.de accessed on March 2018). The FE-mesh reaches from Danube-km 2,583 up to Danube-km 2,557. The FE-mesh was revised by Aquantec in order to make the mesh suitable for the program system HDWAM. A part of the FE-mesh was cut out, from Danube-km 2,581.43 (downstream the barrage Böfinger Halde) up to Danube-km 2,574.67 (downstream the barrage Leibi). The revised FE-mesh includes the floodplain which is flooded in case of a HQ_20_. The part of the FE-mesh used for simulations with the program HDWAM consists of 20,039 knots and 29,742 elements.

The dispersal of different ARB and ARGs is simulated with the 2D-hydraulic approach of HDWAM for steady state scenarios ranging from low water level (gauge Neu-Ulm 22 m^3^/s), medium water level (124 m^3^/s) up to more or less an HQ_20_ (994 m^3^/s) flood. Depending on the flow conditions the dispersal stays in the riverbed itself or extends to the floodplain.

### Statistical evaluation

Box plot graphs were chosen to illustrate the distribution of the measured values using the median values and the quartiles. Therefore, the median values of each sampling campaign were used, resulting in four median values. For the statistical analyses these values were used to calculate the different *p*-values to show significant differences between the treatments. In order to decide which statistical test should be used for determining the significance the data were first analyzed for their normal distribution using the Shapiro-Wilk test. In most of the cases the values for the different detected targets were normally distributed. Therefore, the *t*-test was applied to demonstrate the significance, which is also present with the illustrated figures. In some cases the data were not normally distributed and therefore the Mann-Whitney test was used to indicate significant differences between the samples.

## Results and discussion

### Conventional wastewater treatment and its impacts on facultative pathogenic bacteria and ARGs

**Figure 1:**
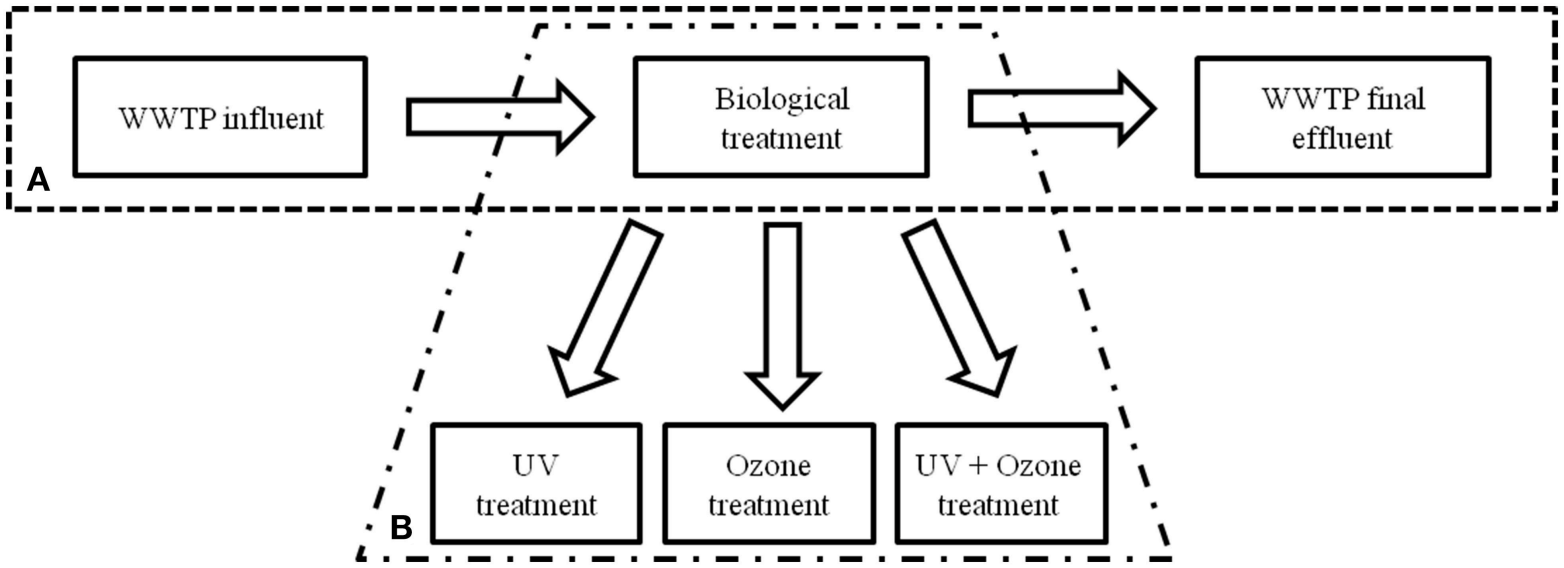
Schematic illustration of the WWTP processes performed at the WWTP under investigation. **(A)** Conventional treatment with biological treatment (activated sludge and sedimentation tank) and **(B)** installation of semi-industrial advanced technologies.

To determine the occurrence of facultative pathogenic bacteria and ARGs during the conventional wastewater treatment process at the WWPT volume based qPCR data were analyzed at three processing steps. Samples of the influent, activated sludge treatment in combination with sedimentation (biological treatment), and the final effluent were under investigation, firstly (Figure 1A).

The abundances of specific marker genes representing specifically facultative pathogenic bacteria and ARGs within the population were normalized to 100 mL wastewater volumes. The used primer sequences are listed in Supplementary Information Table 1. Quality controls were performed as described previously. The selection of the facultative pathogenic bacteria reflects their clinical relevance and their association with wastewaters. There is no regulation or guideline for the presence of such bacteria in municipal wastewaters in Germany, but for other European countries. The regulations of Spain, Cyprus, France, Greece, and Italy have selected *Escherichia coli* as a surrogate for facultative pathogenic bacteria, where also coliforms were studied previously in contaminated waters (Ashbolt et al., 2001). Nevertheless, it became obvious that some facultative pathogenic bacteria like *P. aeruginosa* released by WWTPs did not behave like indicator bacteria in susceptibility for oxidative treatment and regrowth capacities in downstream aquatic environments (Lüddeke et al., 2015; Alexander et al., 2016). Therefore, the following taxonomic marker genes [*16S rRNA* (*Eubacteria*), *23S rRNA* (enterococci), *ecfX* (*P. aeruginosa*), and *yccT* (*E. coli*)] were used for quantification via qPCR. In addition six ARGs (*mecA* (methicillin resistance gene), *ctx-M32* (β- lactame resistance gene), *ermB* (erythromycine resistance gene), *bla*_TEM_ (β- lactame resistance gene), *sul1* (sulfonamide resistance gene), *vanA* (vancomycin resistance gene), and *intI1* (Integrase1 gene) were used to quantify the load factor at the mentioned sampling points of the conventional WWTP. These antibiotic resistance genes were chosen due to their different occurrence in WWTPs (Hembach et al., 2017). The frequently found antibiotic genes (e.g., *bla*_TEM_, *ermB, sul1*, and *intI1*) are suitable tools to show the reduction efficiencies of the different treatment steps. Furthermore, less frequently detected genes were included into the analysis to see if these genes will be effectively reduced during advanced treatments or if they will be still present after the treatments. These used gene targets are considered as suitable parameters for wastewater quality (Berendonk et al., 2015).

**Figure 2:**
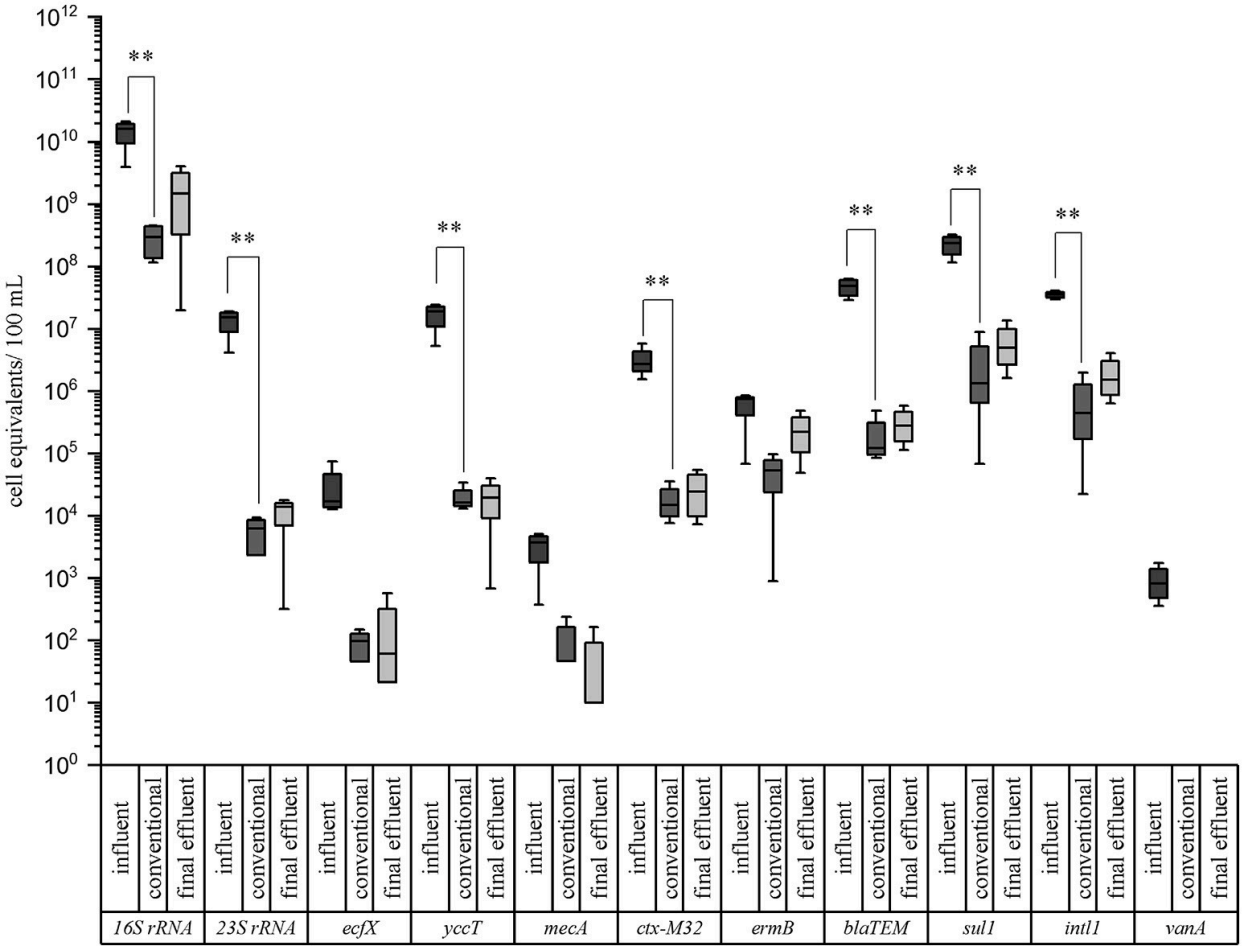
Box plot graphs of the qPCR analyses targeting taxonomic and antibiotic resistance gene markers in wastewater samples of a municipal WWTP. Data are given for the influent, conventional (activated sludge with sedimentation), and effluent samples. Median values, standard deviations, and minimum/maximum values from 4 sampling periods are given. Significance is given by *t*-test calculation and is shown by asterisks (*t*-test; ^**^p < 0.05, ^*^p < 0.1).

The results are illustrated in box plot graphics with medians, standard deviations, and minimum/maximum values of four sampling periods (Figure 2). Median values of the cell equivalents were used for the calculations of the reduction efficiencies. In all cases the measured cell equivalents per 100 mL were highest in the influent samples of the WWTP. A reduction due to the conventional treatment ranging from 1.1 to 3.4 orders of magnitudes (log units) can be observed for all of the tested taxonomic and resistance genes. In case of the taxonomic marker genes the highest reduction was measured for enterococci with 1.51 × 10^7^ cell equivalents/100 mL in the inflow to 6.27 × 10^3^ cell equivalents/100 mL after the conventional treatment (i.e., 3.4 log units reduction). The lowest reduction was observed for *P. aeruginosa*. Here, a reduction of only 2.2 logs, from 1.70 × 10^4^ cell equivalents/100 mL to 9.89 × 10^1^ cell equivalents/100 mL was analyzed. The abundance of *E. coli* was decreased from 1.88 × 10^7^ to 1.64 × 10^4^ cell equivalents/100 mL after the conventional treatment, resulting in a reduction of 3.1 logs. No significant differences occurred between the conventional treatment and the final effluent.

In case of the ARGs, the highest reduction was determined for ß-lactamase gene *bla*_TEM_ (2.6 log units) and vancomycin resistance gene *vanA* (2.9 log units; < LOD), which was not detectable after conventional treatment. More specifically, the β-lactame resistance gene (*bla*_TEM_) was reduced from 4.82 × 10^7^ cell equivalents/100 mL in the influent to 1.22 × 10^5^ cell equivalents/100 mL after the conventional treatment. The *ctx-M32* and *sul1* resistance genes were reduced from 2.73 × 10^6^ to 1.50 × 10^4^ and from 2.35 × 10^8^ to 1.33 × 10^6^ cell equivalents/100 mL after conventional treatment, respectively. The lowest reduction showed *ermB* gene, coding for the erythromycin resistance, with 1.1 log units. Here, the abundance was decreased from 7.51 × 10^5^ cell equivalents/100 mL in the influent to 5.37 × 10^4^ cell equivalents/100 mL after the conventional treatment. Significant differences between the influent and the conventional treatment (*t*-test; ^**^p < 0.05, ^*^p < 0.1) could be calculated for these mentioned genes showing no differences in their significance using the student's *t*-test or the Mann-Whitney test in case of not normally distributed data. Also no significant differences were observed between the conventional treatment and the final effluent. Furthermore, it became obvious that the *P. aeruginosa* gene marker (*ecfX*) and some antibiotic resistance genes *mecA*, and *ermB* were not significantly reduced by the biological treatment using the student's *t*-test. Using the Mann-Whitney test *ecfX* and *mecA* showed a significant reduction. The vancomycin resistance gene, directed against an antibiotic of last choice, was only detected in the influent samples. Nevertheless it became evident that the activated sludge with sedimentation didn't increases the abundances of facultative pathogenic bacteria as well as ARGs. Furthermore the abundances of the gene markers didn't changed significantly from the outflow of the biological treatment to the effluent sampling point. Comparing our data with a previous study of Czekalski et al. (2012), similar cell equivalents per 100 mL or gene copies were measured for the *16S rRNA* representing the total bacterial community and the *sul1* gene coding for the sulfonamide resistance. Other studies like Munir et al. (2011), and Alexander et al. (2015) revealed some differences in gene abundances. These differences may arise from several points, like regional differences, influences of industries and hospitals on the WWTP, as well as different wastewater treatment processes at the WWTPs.

Based on the collected qPCR data showing the presence of facultative pathogenic bacteria marker genes and ARGs in the final effluent of the WWTP (Figure 2), the cell equivalents per 100 mL were converted into cell equivalents per m^3^. For the calculations of the daily charges via the WWTP effluent, these values were multiplied with the annual mean discharge of 1.165 m^3^/s resulting in the amount of released cell equivalents per second and afterwards multiplied with 86400 s to obtain the amount of cell equivalents released within 24 h (Table 1). Furthermore, calculations regarding the dilution factor of different water level scenarios of the receiving river Danube were performed using the obtained cell equivalent per m^3^ data and flow rates of the river for low, mean, and flood waters (Table 1). Furthermore, the calculation of the distribution and dilution within the receiving system allows estimating these risks of dissemination of facultative pathogenic bacteria and antibiotic resistances in downstream bulk water systems used for possible water reuse processes including drinking water conditioning. More specifically, the consideration of scenarios like flood water events are important where facultative pathogenic bacteria and ARGs may be discharged into floodplains and will be further spread into the environment.

**Table 1:** Daily load situation of a municipal wastewater treatment plant effluent.

		**Bacterial concentration at the WWTP**		**Bacterial concentration within the river at different water levels**		
		**Daily discharges (24 h)**	**Discharge per second**	**Low water (Q22)**	**Mean water (Q124)**	**Flood water (HQ20)**
				**(22 m^3^/s)**	**(124 m^3^/s)**	**(994 m^3^/s)**
	**Gene**	**[Cell equivalents/24 h]**	**[Cell equivalents/m**^3^**]**	**[Cell equivalents/m**^3^**]**	**[Cell equivalents/m**^3^**]**	**[Cell equivalents/m**^3^**]**
Eubacteria	*16S rRNA*	1.49E+18	1.72E+13	7.84E+11	1.39E+11	1.73E+10
Enterococci	*23S rRNA*	1.40E+13	1.62E+08	7.36E+06	1.31E+06	1,63E+05
*P. aeruginosa*	*ecfX*	6.19E+10	7.16E+05	3.26E+04	5.78E+03	7.21E+02
*E. coli*	*yccT*	1.97E+13	2.28E+08	1.04E+07	1.84E+06	2.30E+05
Cefotaxime resistance gene	*ctx-M32*	2.49E+13	2.88E+08	1.31E+07	2.32E+06	2.89E+05
Erythromycine resistance gene	*ermB*	2.22E+14	2.57E+09	1.17E+08	2.08E+07	2.59E+06
β- Lactame resistance gene	*bla_*TEM*_*	2.80E+14	3.24E+09	1.47E+08	2.61E+07	3.26E+06
Sulfonamide resistance gene	*sul1*	4.97E+15	5.76E+10	2.62E+09	4.64E+08	5.79E+07
Integrase 1 gene	*intI1*	1.54E+15	1.78E+10	8.10E+08	1.44E+08	1.79E+07

*Shown are the calculated cell equivalents/24 h of the wastewater treatment effluent, as well as the calculated cell equivalents/m^3^ at different water levels for the measured parameters*.

Table 1 describes the 24 h discharges with the highest calculated values for *Eubacteria* as a marker gene for all bacteria followed by *E. coli* and enterococci in a similar range of 10^13^ orders of magnitude present in the WWTP effluent. *P. aeruginosa* was calculated with 2 orders of magnitudes less (10^11^ log units). In case of the ARGs the daily loads range from 10^10^ order of magnitudes for the methicillin resistance gene to 10^15^ log units for the sulfonamide resistance gene. The class-1 specific integron gene *intI1* representing a mobile genetic element for resistance genes was also found to be present in high abundances of 10^15^ log units. The vancomycin resistance gene (*vanA*) was not detected in the final effluent of the WWTP and is therefore not listed in Table 1. Within the river system dilution effects could be calculated. In case of low water events, a dilution effects up to 1.3 orders of magnitude could be calculated. For mean water, and flood water these dilution effects reached values of 2.1 and 3.0 log units, respectively.

With the help of the real quantification data from qPCR analyses and the load calculation equations (see chapter 2.6) the burden of one rivers system impacted by only one WWTP became visible. This calculation did not reflect the already present charges with facultative pathogenic bacteria and antibiotic resistance genes from upstream scenarios, where other entries from additional WWTPs or rain overflow basins at heavy rain seasons impacts the microbial quality of the river system. In consequences, the real burden with facultative pathogenic bacteria and ARGs are expected to be higher even at flood scenarios.

### Impact of advanced wastewater treatment technologies on facultative pathogenic bacteria and ARGs

**Figure 3:**
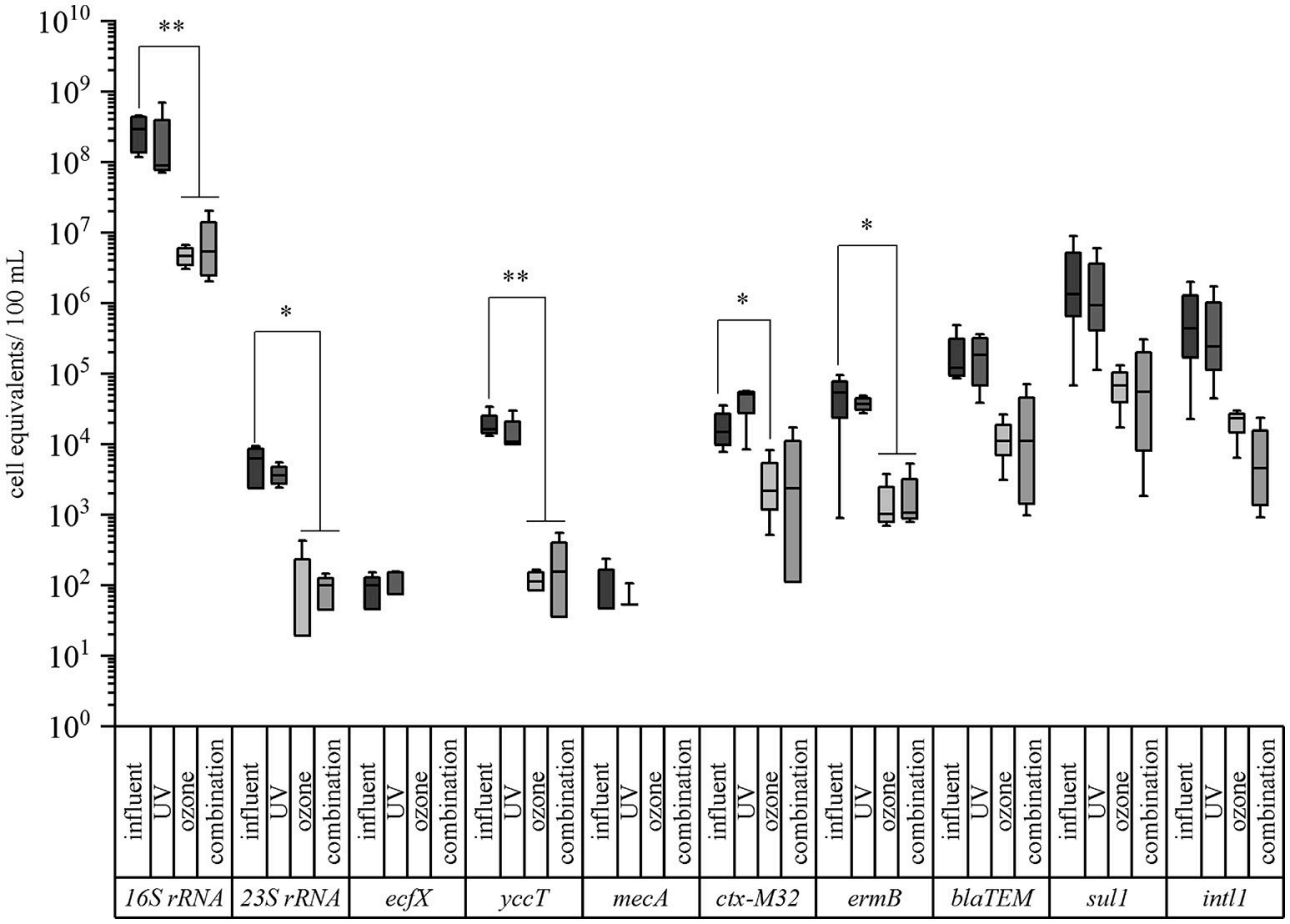
Box plot graphs of the qPCR analyses targeting taxonomic and antibiotic resistance gene markers in advanced treated wastewater samples of a municipal WWTP. Data are given for the conventional treatment (activated sludge with sedimentation, influent), UV treated samples (400 J/m^2^), ozone treated samples (1 g ozone/ g DOC), and the combined treatment of UV and ozone (combination). Median values, standard deviations, and minimum/maximum values from 4 sampling periods are given. Significance is given by *t*-test calculation and is shown by asterisks (*t*-test; ^**^p < 0.05, ^*^p < 0.1).

Different advanced wastewater treatment technologies, i.e., UV irradiation, ozone treatment, and the combination of UV with ozone treatment on conventionally treated wastewater (after activated sludge with sedimentation) were under investigation (Figure 1B). Here, the same taxonomic and antibiotic resistance gene markers were used for qPCR analyses (Supplementary Information Table 1). The vancomycin resistance gene (*van*A) was not analyzed because of its absence after conventional treatment. The biological treated wastewater, i.e., activated sludge treatment followed by sedimentation, was used as reference value (control) for the different reduction efficiencies during the advanced wastewater treatments. In Table 2 the median values calculated for the box plot graph (Figure 3) were used to determine the reduction efficiencies of the different treatment technologies.

**Table 2:** Reduction efficiencies of advanced wastewater treatment technologies on taxonomic and antibiotic resistance gene markers.

**Target**	**Control**	**UV treatment**		**Ozone treatment**		**Combination**	
	**Absolute abundance**	**Absolute abundance**	**Reduction (–)**	**Absolute abundance**	**Reduction (–)**	**Absolute abundance**	**Reduction (–)**
			**Increase (+)**		**Increase (+)**		**Increase (+)**
	**[Cell equivalents/ 100 mL]**	**[Cell equivalents/ 100 mL]**	**[%]**	**[Cell equivalents/ 100 mL]**	**[%]**	**[Cell equivalents/ 100 mL]**	**[%]**
*16S*	2.94E+08	9.04E+07	−69.3%	4.65E+06	−98.4%	5.47E+06	−98.1%
*23S*	6.27E+03	3.61E+03	−42.4%	1.91E+01	−99.7%	9.92E+01	−98.4%
*ecfx*	9.89E+01	7.50E+01	−24.1%	0.00E+00	< LOD	0.00E+00	< LOD
*yccT*	1.50E+04	1.09E+04	−27.4%	1.14E+02	−99.2%	1.57E+02	−99.0%
*mecA*	4.70E+01	0.00E+00	< LOD	0.00E+00	< LOD	0.00E+00	< LOD
*ctxM32*	1.50E+04	5.05E+04	236.3%	2.17E+03	−85.5%	2.38E+03	−84.1%
*ermB*	5.37E+04	3.75E+04	−30.2%	1.01E+03	−98.1%	1.07E+03	−98.0%
*bla*_TEM_	1.22E+05	1.83E+05	50.1%	1.10E+04	−91.0%	1.12E+04	−90.8%
*sul1*	1.33E+06	9.33E+05	−29.9%	6.83E+04	−94.9%	5.53E+04	−95.8%
*intl1*	4.42E+05	2.43E+05	−44.9%	2.34E+04	−94.7%	4.61E+03	−99.0%

*The abundances and reduction efficiencies of conventional treated wastewater (control), UV treated wastewater at 400 J/m^2^ (UV treatment), ozone treated wastewater with 1 g ozone/g DOC (ozone treatment) and the combination of UV and ozone treatment (combination) are illustrated*.

In case of the taxonomic marker genes all three facultative pathogenic bacteria were detectable after conventional treated wastewater. The abundance of the viable fraction after PMA treatment ranged from 9.89 × 10^1^ cell equivalents per 100 mL for *P. aeruginosa* (*ecf* X) to 1.50 × 10^4^ cell equivalents per 100 mL for *E. coli* (*yccT*). The abundance of enterococci (enterococci specific 23S rRNA) and the overall bacterial load (16S rRNA) were determined with 6.27 × 10^3^ cell equivalents per 100 mL and 2.94 × 10^8^ cell equivalents per 100 mL, respectively (Figure 3). In case of the antibiotic resistance genes, the measured cell equivalents per 100 mL ranged from 1.33 × 10^6^ cell equivalents per 100 mL for *sul1* to 1.50 × 10^4^ cell equivalents per 100 mL for *ctx-M32*. The abundances of intI1, *bla*_TEM_ and *ermB* showed values of 4.42 × 10^5^, 1.22 × 10^5^, and 5.37 × 10^4^ cell equivalents per 100 mL, respectively. The abundance of the methicillin resistance gene was determined with 4.70 × 10^1^ cell equivalents per 100 mL. As reference for the determination of the reduction efficiencies of the different treatments the conventional treated wastewater was taken into consideration.

UV treatment resulted in a reduction of the abundance of all taxonomic marker genes ranging from 24.1, 27.4, 42.4, to 69.3% for *P. aeruginosa, E. coli*, enterococci, and 16S rRNA gene, respectively (Table 2, Figure 3). Similar reduction efficiencies were detectable for *sul1, ermB*, and *intI1* showing reduction efficiencies of 29.9, 30.2, and 44.9%, respectively. The cell equivalents per 100 mL were reduced to 9.33 × 10^5^, 3.75 × 10^4^, and 2.43 × 10^5^, respectively. In contrast the antibiotic resistance genes *bla*_*TEM*_ and *ctx-M32* showed an increase in their abundance after the UV treatment. No significant differences could be calculated neither with the student's *t*-test nor with the Mann-Whitney test between the influent samples and the UV treated samples.

UV treatment referring to wastewater treatment technologies seems not to be very effective. Also other studies report that reduction efficiencies could vary between 0.5 and 3.0 log units of gene copies/ 100 mL depending on the used fluences, as well as on the investigated resistance genes. It is reported that *tetA* and *ampC* genes are more resistant to UV treatment compared to *mecA* or *vanA* resistance genes (McKinney and Pruden, 2012). Furthermore, the complex wastewater matrix could influence the reduction efficiencies due to the high turbidity of the wastewater samples so that the UV light cannot interpenetrate the wastewater (Zhuang et al., 2015).

Ozone treatment resulted for all tested taxonomic marker genes in reduction efficiencies between 98.4% in case of the 16S rRNA gene to below the detection limit. *E. coli* and enterococci showed reductions of their abundance of 99.2% to 1.14 × 10^2^ cell equivalents per 100 mL and of 99.7% to 1.91 × 10^1^ cell equivalents per 100 mL. In case of *P. aeruginosa* with a relative low burden at the reference point (after biological treatment) qPCR measures were below the detection limit (Table 2, Figure 3). The ozone treatment showed for all tested antibiotic resistance genes reductions ranging from 85.5 to 98.1%. The methicillin resistance gene (*mecA*) wasn't detectable after the ozone treatment. The strongest reduction was measured for the erythromycin resistance gene (*ermB*) by 98.1% to 1.01 × 10^3^ cell equivalents per 100 mL. The sulfonamide resistance gene (*sul1*) was reduced to 6.83 × 10^4^ cell equivalents per 100 mL resulting in a reduction of 94.9% followed by the integrase 1 gene (*intI1*) with a reduction in percentage of 94.7%. The abundance of the ß-lactame resistance gene (*bla*_*TEM*_) was reduced to 1.10 × 10^4^ cell equivalents per 100 mL (reduction of 91%). The abundance of the cefotaxime resistance gene (*ctx-M32*) showed a reduction of its abundance to 2.17 × 10^3^ cell equivalents per 100 mL (reduction of 85.5%). Significant differences between the influent and the ozone treated wastewater could be calculated with the student's *t*-test for all tested parameters except the enterococci specific marker gene (*23S rRNA*) gene and the erythromycin resistance gene (*ermB*). Here, the data were not normally distributed and the Mann-Whitney test was applied for statistical analysis.

The ozone treatment was able to reduce all the investigated antibiotic resistance genes. In contrast to the chemical micro-pollutants, which are discussed to become reduced to 80% during ozone treatment, microbiological hazardous contamination should be reduced to percentages of at least 99% to avoid any regrowth, afterwards. An advantage of the ozone treatment is it's applicability to microbiology reduction or elimination in parallel with the reduction or transformation of micro-pollutants. It has to be stated that the disinfection efficiency of ozone depends on the ozone concentration, the contact time, and water quality. Especially, dissolved organic carbon (DOC), suspended solids (SS), and particulate matter from activated sludge should be considered during ozonation (Lazarova, 2013; Czekalski et al., 2016; Pak et al., 2016). The used hydraulic retention time of the wastewater was arranged with 5 min. Both, ozone concentration and hydraulic retention time are parameters with could be adapted to increased elimination impacts on bacteria carrying antibiotic resistance genes. In this context unwanted chemical by-products like bromide should not become transformed by elevated ozone concentrations as previously mentioned (von Gunten and Hoigne, 1994; von Gunten, 2003; Lee and von Gunten, 2010).

In addition, the potential mutation of DNA after ozone exposure and toxic transformation products (e.g., bromate and nitrosamines) should be noted. Biological filtration with sand or activated charcoal is frequently recommended after ozonation to avoid the release of newly transformed unwanted compounds to the downstream environments. But, these filter systems bear the risk of microbial regrowth of facultative pathogenic bacteria or ARGs. Hence the ozone treatment should become adjusted to remove bacterial loads in sufficient high efficiencies.

The combination of UV and ozone treatment also revealed high percentages of reduction for all tested bacteria. The relative abundance of *E. coli* could be reduced from 1.50 × 10^4^ cell equivalents per 100 mL to 1.57 × 10^2^ cell equivalents per 100 mL and enterococci were reduced from 6.27 × 10^3^ to 9.92 × 10^1^ cell equivalents per 100 mL, resulting in 99.0 and 98.4% reduction of these bacteria within the surviving population. The eubacterial fraction (16S rRNA gene) was reduced by 98.1% and *P. aeruginosa* again was not detectable after the combined treatment (Table 2, Figure 3). Also the combination of UV and ozone treatment led to a reduction for all tested antibiotic resistance genes from 84.1% up to 99.0%. Here, the abundance of the integrase 1 gene (*intI1*) could be detected with 4.61 × 10^3^ cell equivalents per 100 mL resulting in 99.0% reduction. The erythromycin resistance gene (*ermB*) was reduced to 1.07 × 10^3^ cell equivalents per 100 mL (98.0% reduction) followed by the sulfonamide resistance gene (*sul1*), which was detected with an abundance of 5.53 × 10^4^ cell equivalents per 100 mL resulting in 95.6% reduction. The ß-lactame resistance gene (*bla*_*TEM*_) showed a reduction of 90.8% with a detectable abundance of 1.12 × 10^4^ cell equivalents per 100 mL. The abundance of the cefotaxime resistance gene (*ctx-M32*) was detected with 2.38 × 10^3^ cell equivalents per 100 mL resulting in a reduction of 84.1%. The methicillin resistance gene (*mecA*) wasn't detectable after the combined treatment.

Significant differences between the influent and the UV and ozone treated wastewater could be calculated with the student's *t*-test for all tested parameters except for the erythromycin resistance gene (*ermB*). Here, the data were not normally distributed and the Mann-Whitney test was applied for statistical analysis.

The combination of UV and ozone treatment under the given conditions didn't result in a more effective reduction compared to ozone treatment. This might be due to the particulate material which might be still present after the ozone treatment so that the UV light was not able to interpenetrate the ozone treated wastewater. It would be possible that at further processing steps (e.g., after particle removal via filtration steps) the UV treatment might be a very suitable method to eliminate the residual contaminations. In consequence, adjustments to ozone treatment which achieve a high elimination rate of ARBs and ARGs should have high priority for the application in WWTPs. As mentioned before ozone contact times with an adapted hydraulic retention time at the ozone facility might a possible way to increase the elimination rates.

As previously described ozone treatment is based on radical ion production. Hence, ozone could also induce oxidative stress responses in surviving wastewater populations. It is known, that the impact of ozone given to wastewaters depends on many biotic and abiotic factors like bacteria densities, chemical load, and also suspended solids concentration. This implicates that sub-lethal effects on bacteria can occur promoting stress responses, population shifts, and bacterial selection processes. Dwyer et al. (2009) described the formation of reactive oxygen species (ROS) impacting the metabolism of bacteria. The triggered SOS response contributed to resistance development and the adaptation process would account for an increased robustness toward ROS of affected bacteria. Furthermore, the presence of anti-oxidative mechanisms in different species may lead also to different dynamics in the reduction efficiency of oxidative treatments (Dwyer et al., 2009; Alexander et al., 2016). The efficiencies of the different advanced treatment processes might also depend on the microorganisms carrying the mentioned antibiotic resistance genes. The presence of the genes are not limited to one specific bacterium, but can also be transferred to other so far uncharacterized bacteria from the wastewater population. Therefore, it's difficult to estimate the accessibility of disinfectants (ozone) or physical measurements (UV) on mixed communities in natural habitats. Most of the analyzed ARGs are located on mobile genetic elements described for horizontal gene transfer (HGT). Other studies have shown, that there is a secondary effect of bactericidal antibiotics besides their drug target-specific interaction within bacteria (Kohanski et al., 2007, 2010). There, sub-lethal concentrations of bactericidal antibiotics were used to stimulate the formation of intra-cellular, highly reactive hydroxyl radicals, which contribute to the killing efficiency of bactericidal antibiotics. The induction of oxidative stress by bactericidal antibiotics may induce sub-lethal stress response mechanisms in bacteria that deal not only with the adaptation to the original drug target (antibiotic resistance development), and oxidative damage-associated responses (e.g., *rec*A response). Bacteria which experienced these stress signals, responded, and survived. Therefore, they have a considerable advantage in surviving oxidative wastewater treatments (Alexander et al., 2016). In consequence, higher ozone concentration as proposed to increase the biocidal impacts during advanced wastewater treatment might a good strategy to avoid sub-lethal or selective side effects of ozone in certain bacteria of wastewater populations. Here, we focused on the absolute abundance of bacteria in 100 mL of wastewater. For visualizing changes of the relative abundance within the surviving population caused by these advanced wastewater treatments a normalization to 100 ng DNA would be possible and was shown in previous poplications of the group (Alexander et al., 2016; Jäger et al., 2018).

### Influence of advanced wastewater treatment technologies on DNA lesions

To investigate the occurrence of DNA lesions after the advanced treatments, different assays were performed. Here, antibody based detection systems against CPDs and 6-4 PPs DNA alterations, as well as PCR elongation experiments were performed (Süß et al., 2009; Kraft et al., 2011).

**Figure 4:**
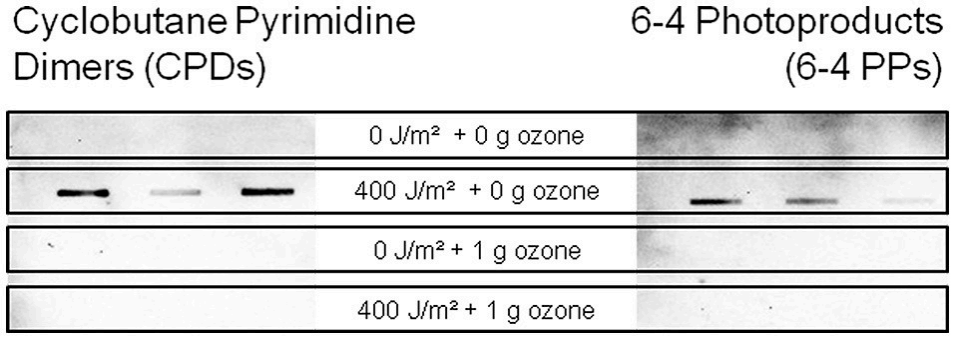
Detection of cyclobutane pyrimidine dimers **(left)** and 6-4 photoproducts **(right)** at 400 J/m^2^ UV and/or 1 g ozone per g DOC with an immunological slot-blot assay.

In case of the antibody based approach, the occurrence of cyclobutane pyrimidine dimers, as well as 6-4 photoproducts in the different treated wastewater samples was analyzed. Here, both DNA lesions could be detected in samples, which were treated with UV intensity of 400 J/m^2^ but neither in the untreated, nor in the samples which were treated with ozone (Figure 4). Increasing the spotted volume of samples which were treated with ozone or a combination of UV and ozone did not result in a detectable signal (data not shown).

To complement the pyrimidine dimer analysis, PCR efficiency experiments with different sized 16S rRNA amplicons were performed according to Süß et al. (2009). In the first sampling campaign the 176 bp amplicon of the 16S rRNA gene showed a reduction of polymerase efficiency compared to the untreated control after UV treatment, whereas for the ozone treatment no PCR efficiency reduction was detectable (Table 3). The combination of UV and ozone treatment showed a small decrease in polymerase efficiency. In case of the 490 bp amplicon, polymerase efficiencies were decreased for all different treatment types. For the 880 bp amplicon the strongest reduction in polymerase efficiency could be detected after the UV treatment and after the combined treatment, whereas ozone didn't lead to a reduction in the PCR efficiency. These results underline the strong impact of UV irradiation on the DNA integrity of bacteria which might impact the mutation rates since 16S rDNA amplicons are representatives of the total bacterial genome. In consequence sub-lethal changes in the DNA integrity might be responsible for newly introduced mutations and might be responsible for bacteria evolution including antibiotic resistance.

**Table 3:** Detection of DNA damages via PCR experiments.

	**Sampling campaign 1**			**Sampling campaign 2**		
	**176 bp amplicon**	**490 bp amplicon**	**880 bp amplicon**	**176 bp amplicon**	**490 bp amplicon**	**880 bp amplicon**
Control	1.0	1.0	1.0	1.0	1.0	1.0
UV treatment	0.62	0.69	0.47	0.21	0.99	0.98
Ozone treatment	1.5	0.76	1.0	0.11	0.71	0.93
Combination	0.92	0.87	0.76	0.1	0.23	0.36

*The quantified light units of the different treatments are normalized to the corresponding amplicon of the conventionally treated wastewater (control). The amplicons were separated by agarose gel electrophoresis and the light units (LU) of each amplicon were determined and normalized to their corresponding amplicon of the untreated wastewater sample*.

The second sampling campaign resulted for the 176 bp amplicon in reduced efficiencies of 0.21, 0.11, and 0.1 for UV, ozone, and the combined treatment, respectively. For the 490 bp amplicon no reduction in efficiency was detectable after UV treatment. After the ozonation and the combination of UV and ozone treatment a reduction of the polymerase efficiency was detectable (0.71 and 0.23). No effects could be seen for the 880 bp amplicon after UV or ozone treatment. Only the combination resulted in a weaker polymerase efficiency of 0.36 (Table 3). In consequence, these DNA lesions occur randomly within different regions of the genome. Therefore, there is some variability in the frequency of occurrence of these DNA lesions within the different amplicons, which has different effects on PCR efficiencies.

The PCR based experiments showed that DNA lesions are present after the combined treatment of UV and ozone, but there are no pyrimidine dimers detectable via the immunological assay. Also in the ozone treated samples no pyrimidine dimers were detected by the chemiluminescence measurements, whereas, DNA alterations were detectable in the PCR efficiency experiments. This might be an effect induced by the ozone reaction with the DNA molecule, which results in other types of DNA lesions compared to UV treatment. It is reported, that the kinetics of ozone molecules are higher for thymine (rate constant 3.4 × 10^4^ L^*^mol^−1^ s^−1^) than for guanine, cytosine, or adenine (Alexander et al., 2016) and that the thymine reacts with the ozone at the position of the methyl group at the C(5)-C(6) double bond, which has a noticeable effect on the rate of reaction (Flyunt, 2007). The oxidation at positions C(5) and C(6) may inhibit the dimer formation and therefore no CPDs and 6-4 PPs were detectable via the immunological assay.

These different degrees of DNA changes induced by UV-irradiation, as well as ozone-treatment especially at sub-lethal levels are known to trigger repair mechanisms in bacteria like *rec*A gene expression (Jungfer et al., 2007), which is a key regulator for recombination events and, therefore, can lead to an increased mutation rate and uptake/incorporation of extracellular DNA. This promotes the HGT, which is one of the main factor in resistome evolution in aquatic habitats (Fall et al., 2007; Aminov, 2011; Chao et al., 2013). Recombination events can also promote adaptation processes as well as the evolution of bacteria and ARGs. Again, elevated ozone concentration or adapted hydraulic retention times might help to suppress these unwanted side-effects in bacteria driving HGT or antibiotic resistance evolution.

### Hydraulic simulations of dispersal of several ARB and ARGs in the danube downstream of WWTP

For three bacteria and three resistance genes listed in Table 4 2D-hydraulic simulations with the Hydrodynamic Wave Propagation Model (HDWAM) have been conducted in order to determine the dispersal of the microbiological parameters. Simulations were done with steady state runoff in the river Danube of 22, 124, and 994 m^3^/s.

**Table 4:** Concentration of bacteria and resistance genes in the outlet of WWTP which were used as input for the simulation with the hydraulic program HDWAM.

**Facultative pathogenic bacteria**	**Outlet WWTP**
	**[cell equival./m^3^]**
*Escherichia coli*	9.20E+08
*Enterococcus spp*.	6.00E+07
*Pseudomonas aeruginosa*	6.43E+06
**Antibiotic resistance genes**	
Sulfonamide resistance gene (*sul1*)	3.20E+10
β- Lactame resistance gene (bla_TEM_)	3.83E+09
Erythromycine resistance gene (*ermB*)	2.88E+09

**Figure 5:**
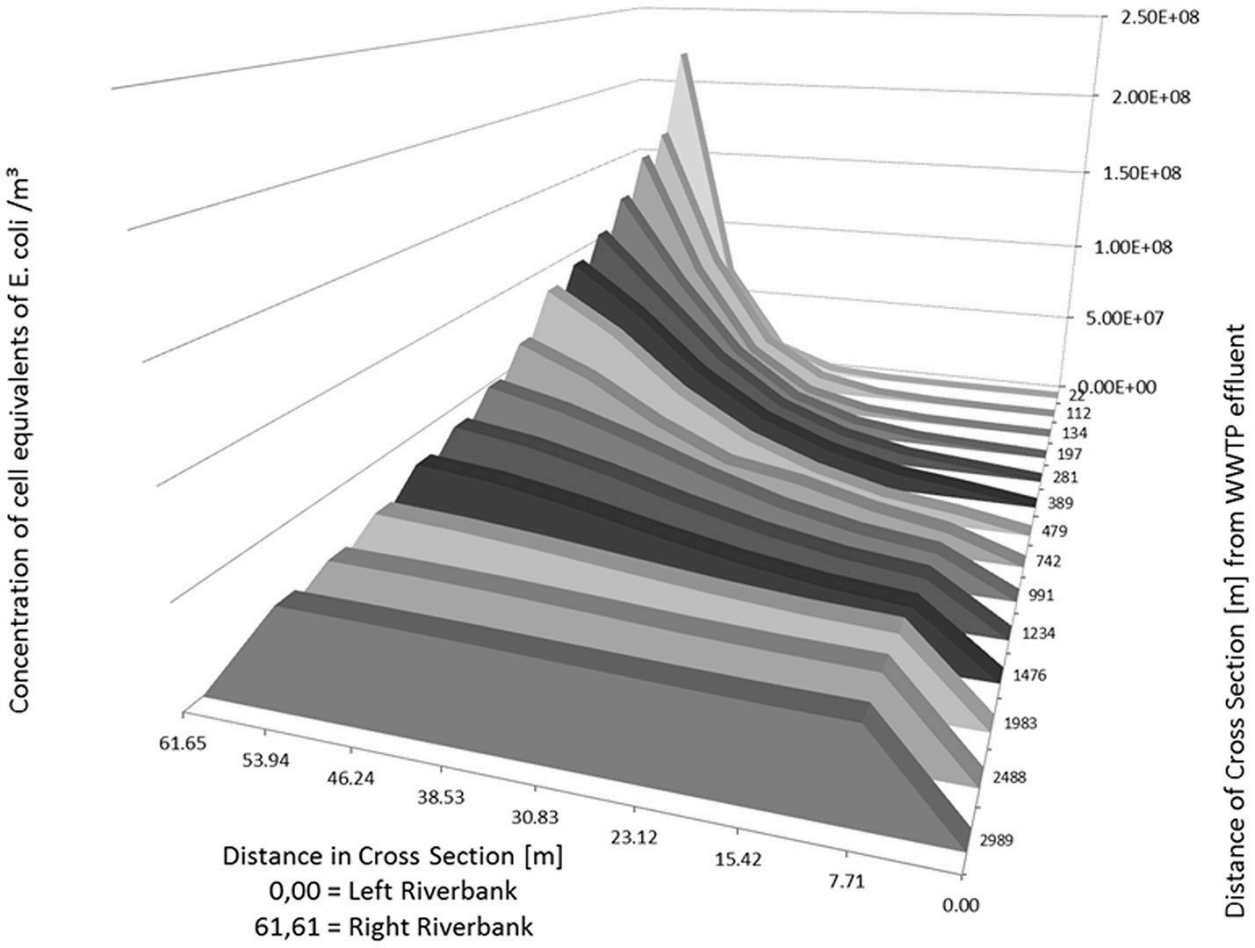
Distribution of calculated (2D-HDWAM) concentration of *E. coli* in the river Danube downstream of the WWTP for different cross sections, 9.20 × 10^8^ cell equivalents/m^3^ in outlet of WWTP, discharge of Danube at 22 m^3^/s (NQ).

**Figure 6:**
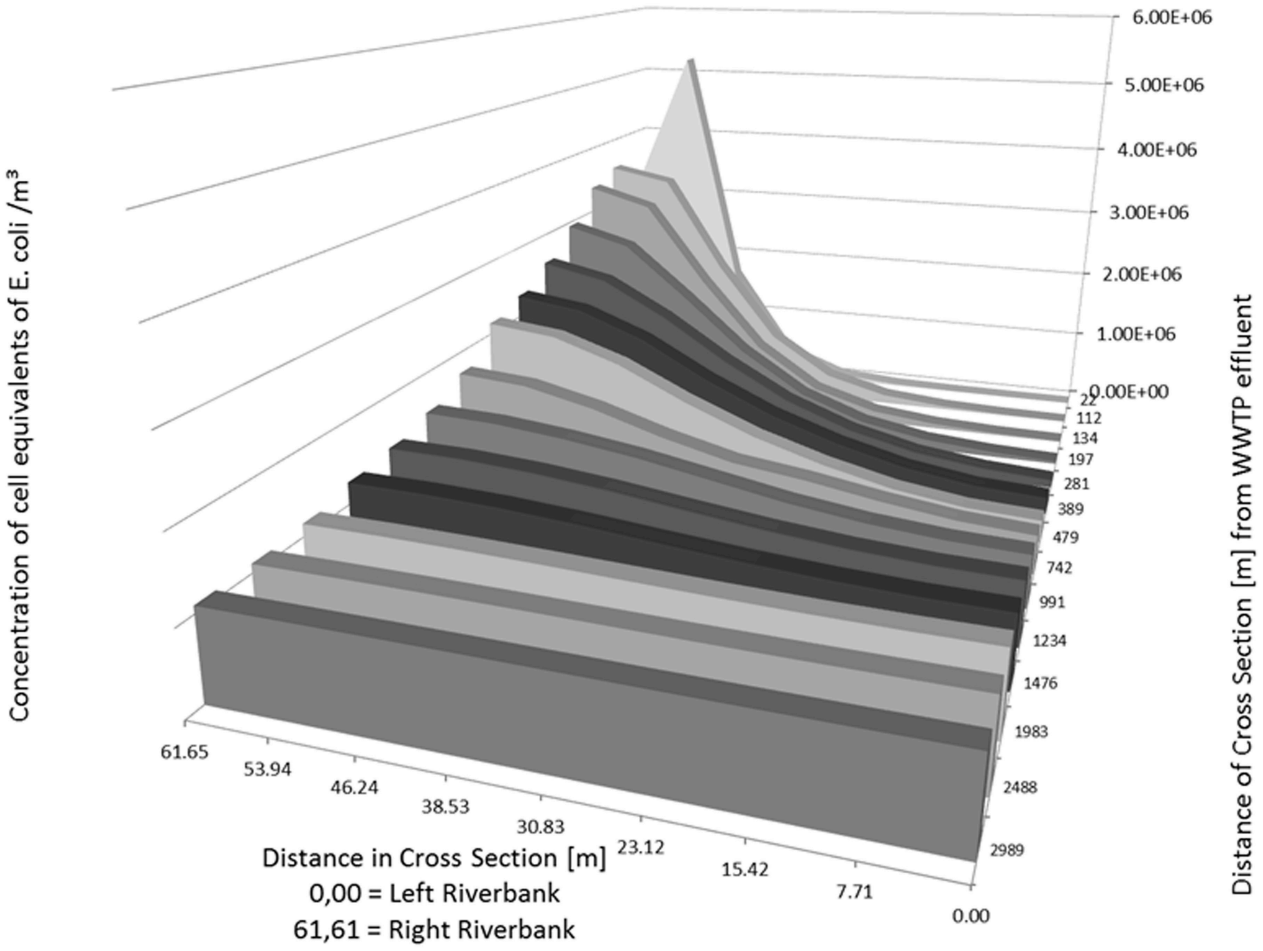
Distribution of calculated (2D-HDWAM) concentration of *E. coli* in the river Danube downstream of the WWTP for different cross sections, 9.20 × 10^8^ cell equivalents/m^3^ in outlet of WWTP, discharge of Danube at 994 m^3^/s (HQ20).

As an example, the Figures 5, 6 show the concentration of *E. coli* at several knots of a cross section of the river Danube from 22 m to about 3,000 m downstream of the outlet of the WWTP. The simulated input from the WWTP is 1.165 m^3^/s with a concentration of *E. coli* in the WWTP outlet of 9.20 × 10^8^ cell equivalents/m^3^. The runoff of the river Danube is simulated with steady state flow conditions of 22 m^3^/s (Figure 5) and 994 m^3^/s (Figure 6).

The runoff of 22 m^3^/s stays in the riverbed itself. The maximum concentration of *E. coli* with a cell equivalent of ~2.21 × 10^8^ is calculated at 22 m downstream of the WWTP. According to the results of the hydraulic model after about 3,000 m downstream of the outlet of WWTP the concentration of *E. coli* is more or less evenly distributed across the river Danube with an average concentration of *E. coli* of about 4.63 × 10^7^ cell equivalents/m^3^.

At a runoff of 994 m^3^/s the maximum concentration is about 5.22 × 10^6^ cell equivalents/m^3^ near the inflow point of the WWTP. The inflow point of the WWTP to the river is situated several meters from the right riverbank toward the left riverbank (Table 5, bold numbers), not directly at the riverbank. Therefore, the concentration at the cross section 22 m is the highest in the point 53.94 m (left riverbank is 0.00 m). Further downstream the cell equivalents mix and in the following cross sections the concentration decreases from the right (61.65 m) to the left river bank (0.00 m) (Table 5 and Figure 6). Similar to the simulation with a runoff of 22 m^3^/s in the river Danube there is a more or less evenly distribution of *E. coli* across the Danube after about 3,000 m with an average concentration of about 1.08 × 10^6^ cell equivalents/m^3^.

**Table 5:** Calculated (2D-HDWAM) concentration of cell equivalents of *E. coli* for cross sections of the river Danube from outlet of WWTP downstream to 3,000 m.

	**Position of cross section across the Danube [m]; inflow point of WWTP situated at right riverbank steady state runoff of Danube at 994 m**^**3**^**/s**									
		**0.00 Left riverbank**	**7.71**	**15.42**	**23.12**	**30.83**	**38.53**	**46.24**	**53.94**	**61.65 Right riverbank**
Downstream distance from WWTP outlet [m]	22	6.956E+02	1.445E+03	6.064E+03	2.373E+04	9.622E+04	4.303E+05	1.681E+06	5.223E+06	2.879E+06
	112	7.215E+03	1.601E+04	4.472E+04	1.272E+05	3.626E+05	9.348E+05	2.024E+06	3.369E+06	3.547E+06
	134	1.310E+04	2.621E+04	6.593E+04	1.664E+05	4.313E+05	1.021E+06	2.005E+06	3.130E+06	3.379E+06
	197	3.854E+04	7.090E+04	1.438E+05	3.102E+05	6.450E+05	1.219E+06	1.984E+06	2.703E+06	2.947E+06
	281	1.087E+05	1.655E+05	2.837E+05	5.042E+05	8.450E+05	1.338E+06	1.913E+06	2.391E+06	2.582E+06
	389	2.236E+05	2.720E+05	4.107E+05	6.418E+05	9.484E+05	1.356E+06	1.843E+06	2.184E+06	2.310E+06
	479	2.936E+05	3.389E+05	4.685E+05	6.858E+05	9.638E+05	1.330E+06	1.756E+06	2.053E+06	2.155E+06
	742	4.961E+05	5.535E+05	6.752E+05	8.265E+05	9.427E+05	1.168E+06	1.444E+06	1.649E+06	1.720E+06
	991	6.701E+05	7.032E+05	7.819E+05	9.057E+05	1.071E+06	1.236E+06	1.362E+06	1.454E+06	1.487E+06
	1,234	7.983E+05	8.189E+05	8.725E+05	9.582E+05	1.068E+06	1.185E+06	1.278E+06	1.335E+06	1.350E+06
	1,476	8.873E+05	9.020E+05	9.404E+05	9.999E+05	1.078E+06	1.161E+06	1.227E+06	1.265E+06	1.275E+06
	1,983	1.002E+06	1.006E+06	1.020E+06	1.044E+06	1.076E+06	1.108E+06	1.135E+06	1.153E+06	1.158E+06
	2,488	1.044E+06	1.046E+06	1.052E+06	1.063E+06	1.078E+06	1.091E+06	1.101E+06	1.107E+06	1.108E+06
	2,989	1.062E+06	1.062E+06	1.065E+06	1.071E+06	1.078E+06	1.085E+06	1.090E+06	1.093E+06	1.093E+06

**Figure 7:**
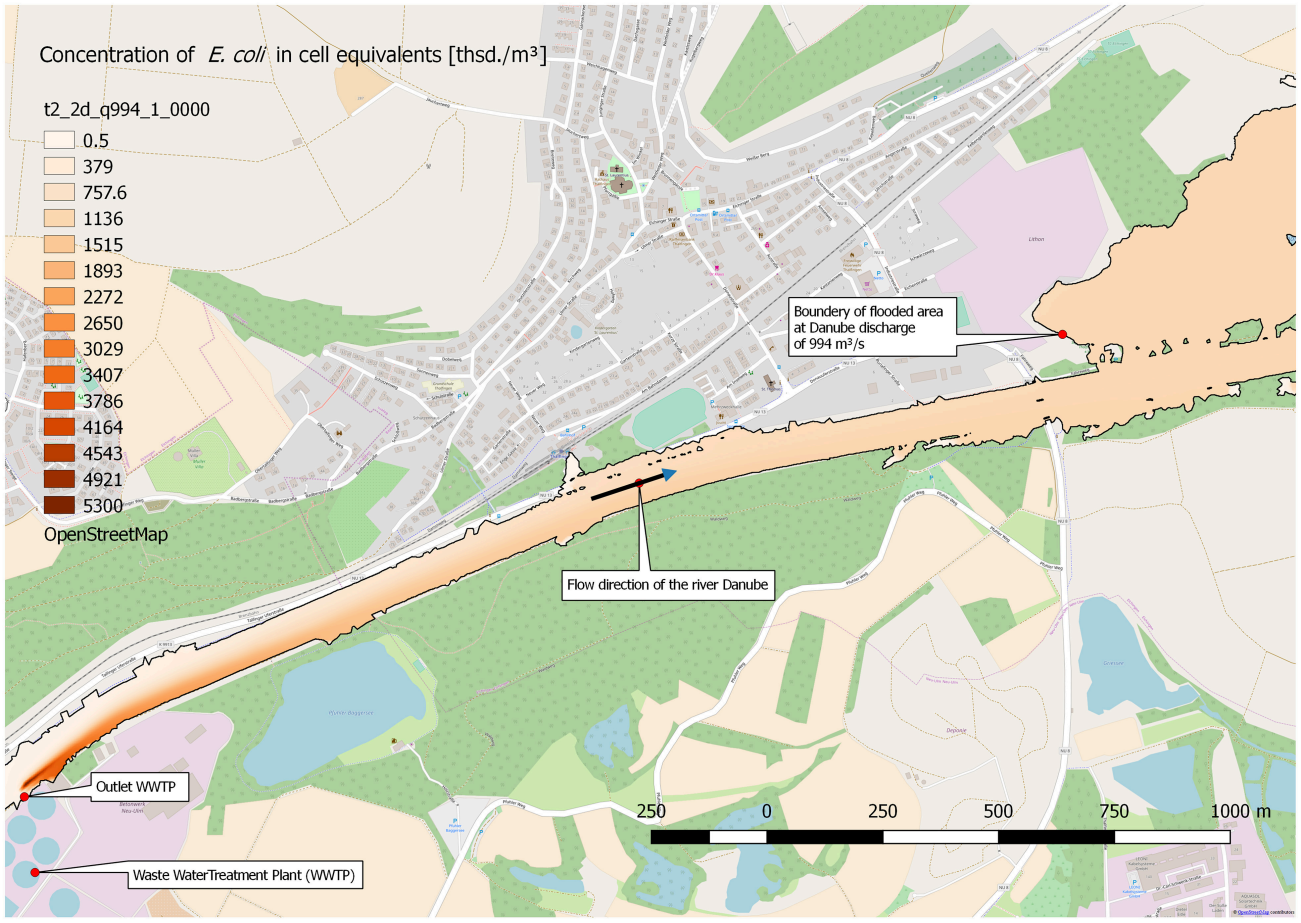
Distribution of calculated (2D-HDWAM) concentration of *E. coli* in the river Danube downstream of the WWTP, 9.20 × 10^8^ cell equivalents/m^3^ in outlet of WWTP, discharge of Danube at 994 m^3^/s (HQ20), interpolated results.

The runoff of 994 m^3^/s in the Danube, and with it *E. coli* with a concentration of about 1.08 × 10^6^ cell equivalents/m^3^, spreads also to parts of the Danube floodplain. Figure 7 shows the maximum extend of the flooding and the concentration of *E. coli* at a steady state runoff in the Danube of 994 m^3^/s. The stretch ranges from the outlet of the WWTP to about 3,500 m downstream. In consequence, the concentration of *E. coli* in the flooded area of the river Danube floodplain is at about 1.08 × 10^6^ cell equivalents/m^3^.

## Conclusion

It was shown that a large WWTP (400.000 p.e.) plays an important part in the distribution of facultative pathogenic bacteria and antibiotic resistances after conventional treatment. The calculation of the daily loads of the WWTP and the consideration of dilution factors of different water level scenarios of the receiving river underline the high burden situations in the adjacent aquatic environment.

Molecular biology analyses revealed that the overall bacterial load and the majority of other clinically relevant bacterial targets were reduced during ozone/UV treatment using semi-industrial facilities, but not eliminated. Antibiotic resistance genes were still found to be present in the effluents under the adjusted parameters within the surviving population. In addition, the occurrence of DNA alterations like CPDs and 6-4 PPs, which were shown to be induced during UV treatment, as well as DNA lesions induced by ozonation might up-regulate specific DNA repair mechanisms like *recA* activities, which are known to enhance horizontal gene transfer, but also mutations rates. Both contribute also to antibiotic resistance evolution and the risk potential in aquatic environments.

Furthermore, the model of the distribution within the river system, which based on data from a conventional working, full-scaled WWTP, showed that a homogenous distribution is achieved after just a few kilometers. The model systems also showed the impacts on downstream river locations used for indirect water reuse or raw water source for drinking water conditioning. Especially at flood water events, facultative pathogenic bacteria and ARGs may be discharged into floodplains. Therefore, it is important to minimize the risk of contamination for the environment and the public health by using advanced treatment technologies to reduce the bacterial load and ARGs at WWTPs.

Further advanced treatment options are also available which may be suitable for reducing the bacterial load in WWTPs like the ultrafiltration. But these technologies might not be able to reduce other micro-pollutants. Therefore, a combination of different methods may lead to an adequate reduction of all types of pollution. Therefore, to the already available guidelines for the removal of chemical pollutants at WWTPs it is necessary to develop additional or adjusted strategies and guidelines adapted for the removal of microbial contaminants in wastewater, including facultative pathogenic bacteria and ARGs.

## Author contributions

TS coordinated and organized the experiment. TJ, NH, and JA performed the experiments and generated the scientific data. CH arranged local support at the municipal wastewater treatment plants, executed sampling procedures, and provided WWTP specific data. AW provided the equipment of advanced treatment techniques. CE and GK carried out the calculations and simulations of the computer-based model.

### Conflict of interest statement

The authors declare that the research was conducted in the absence of any commercial or financial relationships that could be construed as a potential conflict of interest.
