# Supplementary material for: Reduction of Antibiotic Resistant Bacteria During Conventional and Advanced Wastewater Treatment, and the Disseminated Loads Released to the Environment
Source: Front Microbiol. 2018 Oct 30;9:2599. doi: 10.3389/fmicb.2018.02599 (PMC6218952; doi:10.3389/fmicb.2018.02599)
Supplement: Supplementary file 1 [file Table_1.DOCX]

**SI Table 1:** List of all used primer systems with their corresponding target gene, expected amplicon size, accuracy and efficiency of the used calibration curves.

| **Organism** | **Target Gene** | **Primer** | **Sequence** | **Amplicon** | **Accuracy (R²)** | **Efficency [%]** | **Reference** |
| --- | --- | --- | --- | --- | --- | --- | --- |
| **Antibiotic resistance genes** | |  |  |  |  |  |  |
| *E. coli* pNorm* | *sul1* | sul1-FW | CGCACCGGAAACATCGCTGCAC | 163 bp | 0.999 | 97.6 | ([Rocha et al., 2018](#_ENREF_4)) |
|  |  | sul1-RV | TGAAGTTCCGCCGCAAGGCTCG |  |  |  |  |
| *E. coli* pNorm* | *intI1* | intI.1-LC1 | GCCTTGATGTTACCCGAGAG | 196 bp | 1.0 | 94.1 | ([Rocha et al., 2018](#_ENREF_4)) |
|  |  | intI.1-LC5 | GATCGGTCGAATGCGTGT |  |  |  |  |
| *S. aureus+* | *mecA* | mecA1 FP | CGCAACGTTCAATTTAATTTTGTTAA | 91 bp | 1.0 | 99.8 | ([Volkmann et al., 2004](#_ENREF_5)) |
| *CNS* |  | mecA1 RP | TGGTCTTTCTGCATTCCTGGA |  |  |  |  |
| *E. coli* pNorm* | *ctx-M-32* | ctxm32-F | CGTCACGCTGTTGTTAGGAA | 155 bp | 1.0 | 92.5 | ([Rocha et al., 2018](#_ENREF_4)) |
|  |  | ctxm32-R | CGCTCATCAGCACGATAAAG |  |  |  |  |
| *S. hyointestinales* | *ermB* | ermB-F | TGAATCGAGACTTGAGTGTGCAA | 71 bp | 1.0 | 99.7 | ([Alexander et al., 2015](#_ENREF_1)) |
|  |  | ermB-R | GGATTCTACAAGCGTACCTT |  |  |  |  |
| *E. coli* pNorm* | *bla_TEM_* | qblaTEM-F | TTCCTGTTTTTGCTCACCCAG | 112 bp | 0.999 | 100.8 | ([Rocha et al., 2018](#_ENREF_4)) |
|  |  | qblaTEM-R | CTCAAGGATCTTACCGCTGTTG |  |  |  |  |
|  | *vanA* | vanA-For | TCTGCAATAGAGATAGCCGC | 376 bp | 1.0 | 91,6 | ([Klein et al., 1998](#_ENREF_3)) |
|  |  | vanA Rev | GGAGTAGCTATCCCAGCATT |  |  |  |  |
| **Taxonomic marker genes** | |  |  |  |  |  |  |
| *E. coli* | *yccT* | yccTFP | GCATCGTGACCACCTTGA | 59 bp | 0.994 | 98.4 | ([Clifford et al., 2012](#_ENREF_2)) |
|  |  | yccTRP | CAGCGTGGTGGCAAAA |  |  |  |  |
| *P. aeruginosa* | *ecfX* | ecfXRT-F | AGCGTTCGTCCTGCACAAGT | 81 bp | 0.999 | 101.7 | ([Clifford et al., 2012](#_ENREF_2)) |
|  |  | ecfXRT-R | TCCACCATGCTCAGGGAGAT |  |  |  |  |
| *Enterococcus* spp. | 23S rRNA | ECST784F | AGAAATTCCAAACGAACTTG | 93 bp | 1.0 | 90.1 | ([Volkmann et al., 2004](#_ENREF_5)) |
|  |  | ENC854R | CAGTGCTCTACCTCCATCATT |  |  |  |  |
| Eubacteria;  *E. coli* pNorm* | 16S rRNA | 331-F | TCCTACGGGAGGCAGCAGT | 195 bp | 1.0 | 96.6 | ([Rocha et al., 2018](#_ENREF_4)) |
|  |  | 518-R | ATTACCGCGGCTGCTGG |  |  |  |  |

References

Alexander, J., Bollmann, A., Seitz, W., and Schwartz, T. (2015). Microbiological characterization of aquatic microbiomes targeting taxonomical marker genes and antibiotic resistance genes of opportunistic bacteria. *Sci. Total Environ.* 512**,** 316-325.

Clifford, R.J., Milillo, M., Prestwood, J., Quintero, R., Zurawski, D.V., Kwak, Y.I., et al. (2012). Detection of bacterial 16S rRNA and identification of four clinically important bacteria by real-time PCR. *PLoS One.* 7(11)**,** e48558.

Klein, G., Pack, A., and Reuter, G. (1998). Antibiotic resistance patterns of enterococci and occurrence of vancomycin-resistant enterococci in raw minced beef and pork in Germany. *Appl. Environ. Microbiol.* 64(5)**,** 1825-1830.

Rocha, J., Cacace, D., Kampouris, I., Guilloteau, H., Jäger, T., Marano, R.B., et al. (2018). Inter-laboratory calibration of quantitative analyses of antibiotic resistance genes. *Journal of Environmental Chemical Engineering*.

Volkmann, H., Schwartz, T., Bischoff, P., Kirchen, S., and Obst, U. (2004). Detection of clinically relevant antibiotic-resistance genes in municipal wastewater using real-time PCR (TaqMan). *J. Microbiol. Methods* 56(2)**,** 277-286.
